# Circulating protein biomarkers of physical fitness associated with cardiometabolic risk in women after gestational diabetes: a PONCH study

**DOI:** 10.1186/s12933-026-03120-4

**Published:** 2026-02-27

**Authors:** Emilia Kristiansson, Agneta Holmäng, Kristina Wallenius, H. Sophia Chung, Sonja Hess, Stefan Pettersson, Klavs Madsen, Ulrika Andersson-Hall

**Affiliations:** 1 Department of Physiology, Institute of Neuroscience and Physiology, Sahlgrenska Academy, University of Gothenburg, Gothenburg, Sweden; 2 Bioscience Metabolism, Early CVRM, BioPharmaceuticals R&D, AstraZeneca, Gothenburg, Sweden; 3 Centre for Genomics Research, Discovery Sciences, BioPharmaceuticals R&D, AstraZeneca, Gaithersburg, Maryland USA; 4 Centre for Health and Performance, Department of Food and Nutrition, and Sport Science, University of Gothenburg, Gothenburg, Sweden; 5 The Norwegian School of Sports Sciences, Oslo, Norway

**Keywords:** Gestational diabetes, Type 2 diabetes, Cardiometabolic disease, Cardiovascular disease, Physical fitness, Aerobic capacity, Muscle strength, Serum proteomics, Biomarkers

## Abstract

**Background:**

Women with prior gestational diabetes mellitus (GDM) have an elevated risk of developing cardiometabolic diseases, including type 2-diabetes and cardiovascular disease. While physical fitness is protective, circulating molecular biomarkers linking fitness to long-term metabolic health in this population remain poorly understood. This study aimed to identify serum proteins associated with aerobic capacity and muscle strength 10 years after GDM, and explore their biological functions related to cardiometabolic risk.

**Methods:**

We assessed aerobic fitness (VO_2_peak), peak fat oxidation, and maximal isometric muscle strength of five muscle groups in 38 women from the post-GDM PONCH-cohort. Serum proteins were analysed using mass spectrometry-based proteomics. Associations between proteins, fitness variables, and clinical markers were tested using Spearman correlations with FDR correction, and age- and medication-adjusted sensitivity analysis. Group differences across four fitness-level groups (defined by aerobic fitness and muscle strength) and glycaemic status groups were analysed using linear regression models and Kruskal-Wallis tests, with age- and medication-adjusted sensitivity analyses. Exercise responsiveness of selected proteins was assessed in an independent cohort of untrained men undergoing six weeks of supervised aerobic training (*n* = 28), with pre-post changes assessed using Wilcoxon signed-rank tests.

**Results:**

Thirty-five proteins were associated with at least one fitness variable, of which 21 remained significant after age- and medication-adjusted sensitivity analysis. Nine proteins correlated with both VO_2_peak and muscle strength. Identified proteins mapped to key cardiometabolic pathways, including metabolic regulation, immune response, complement activation, oxidative stress, and extracellular matrix remodelling. Five proteins (PON3, IGF1, CRISP3, COL6A3, C3) emerged as particularly interesting, showing the largest and most consistent effect sizes across fitness variables and associations with central adiposity, blood lipids, blood pressure, and insulin resistance. Stratified analysis across the four fitness-level groups identified IGF1, PON3, and PRG4 as markers of higher overall-fitness. In the independent training cohort, nine of the fitness-associated proteins changed following aerobic training without significant weight-loss.

**Conclusions:**

This study identified circulating proteins linked to physical fitness and cardiometabolic health in women after GDM. These findings suggest fitness-associated serum proteins may serve as biomarkers of early metabolic dysfunction and potential targets for exercise-based prevention of T2D and cardiovascular disease.

**Graphical abstract:**

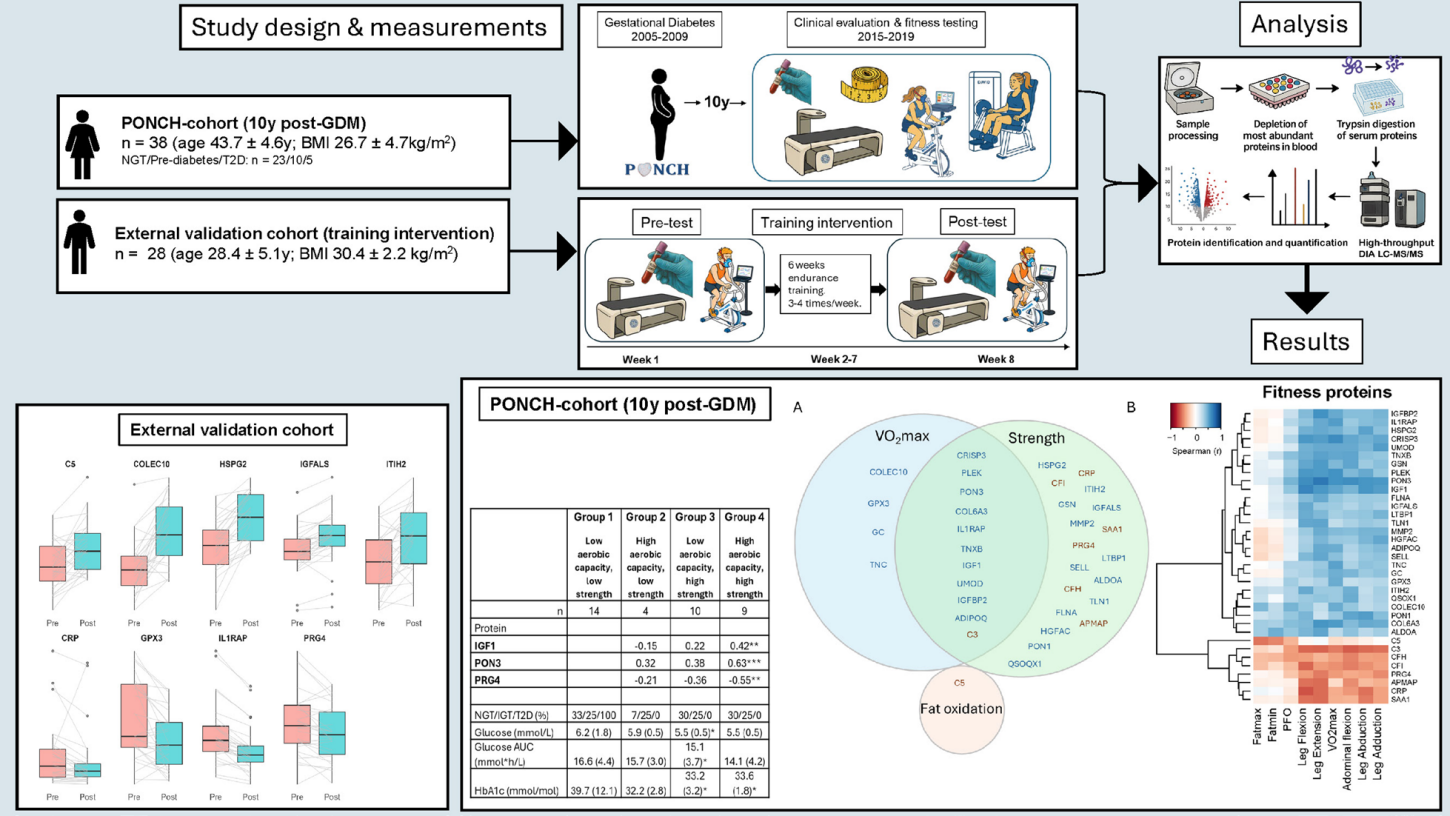

**Supplementary Information:**

The online version contains supplementary material available at 10.1186/s12933-026-03120-4.

## Research highlights

**What is currently known about this topic?**

- Women with prior gestational diabetes mellitus (GDM) have elevated risk of cardiometabolic disease.
- Higher physical fitness improves metabolic health, but molecular fitness biomarkers are less studied.

**What is the key research question?**

- Which circulating proteins are associated with physical fitness 10 years after GDM?

**What is new?**

- This study identifies 35 serum proteins linked to aerobic capacity and muscle strength after GDM.
- Key proteins (PON3, IGF1,CRISP3, COL6A3,C3) consistently related to fitness and metabolic health.
- The proteins may reflect metabolic, inflammatory, and structural pathways in cardiometabolic risk.

**How might this influence clinical practice?**

- Serum fitness proteins may be accessible biomarkers of post-GDM metabolic risk and exercise effect

## Introduction

Gestational diabetes mellitus (GDM), defined as the onset or first recognition of hyperglycaemia during pregnancy, is one of the most prevalent pregnancy complications. Although GDM typically resolves immediately after delivery [1], women with a history GDM have an almost tenfold increased risk of developing type 2-diabetes (T2D) [2], with studies showing up to 30% prevalence within five years after pregnancy [3]. Importantly, T2D development after GDM often represents an early-onset form of the disease and is accompanied by a high cardiometabolic risk burden, including increased risk of cardiovascular disease, hypertension, dyslipidaemia, and premature mortality [4].

T2D is a complex, progressive condition that often develops gradually over many years. By the time T2D becomes symptomatic, chronic complications have often already emerged, underscoring the importance of early detection and understanding of its pathophysiology. Consequently, GDM not only reveals a future risk of disease development but provides an opportunity for early intervention and prevention [2]. Therefore, gaining a comprehensive understanding of the pathophysiological changes that occur after GDM is essential for precise screening, early intervention, and informed medical decision-making.

Regular exercise and moderate-to-high aerobic capacity improve glucose homeostasis, enhance insulin secretion and sensitivity [5], and reduce the risk of T2D [6], and cardiovascular disease [7], as well as mortality [8]. Aerobic exercise increases aerobic capacity [8–11] and positively impacts body composition, blood lipids, immune system, and glucose metabolism [9]. Lower aerobic capacity and muscle strength have been consistently observed in individuals with T2D [12, 13], with a strong inverse linear association between aerobic capacity and cardiometabolic disease [10]. Both low aerobic capacity and reduced muscle strength are linked to increased cardiometabolic risk and adverse cardiovascular outcomes [14]. While muscle strength has been less studied than aerobic capacity, it is clinically relevant for individuals with compromised glucose metabolism. Skeletal muscle, as one of the largest tissues in the human body, accounts for up to 80% of insulin-stimulated glucose uptake [6] and is the primary tissue underpinning physical work capacity and athletic performance [15]. Skeletal muscle demonstrates remarkable adaptation in response to environmental changes such as diet, and physical activity and inactivity [16]. Peak fat oxidation (PFO), measured during incremental/submaximal exercise [17], which is generally higher in aerobically trained individuals, has also been linked to insulin sensitivity. PFO may be influenced by impaired fat oxidation capacity, which contributes to lipid intermediate accumulation and disrupted insulin signalling in oxidative tissues [17]. However, while the benefits between greater aerobic capacity and muscle strength and cardiometabolic health are established, there is limited research on the relationship between the fitness variables and the development of T2D after GDM. Our previous research in the PONCH cohort, which is a unique longitudinal study detailing metabolic phenotyping 6 and 10 years post GDM, showed that high aerobic fitness and in particular high muscle strength strongly correlated with better glycaemic and metabolic outcomes after GDM, independent of BMI [18].

Using cross-sectional data from the PONCH cohort six years post-GDM, we have shown that deep clinical phenotyping combined with NMR-based metabolomics identified distinct metabolic profiles associated with impaired glucose tolerance and progression to T2D [19]. Building on this work, we identified circulating proteomic signatures that either predict development of prediabetes/T2D or are associated with established prediabetes/T2D in women 6–10 years after GDM pregnancies [20]. In the current paper, we extend these findings by integrating comprehensive fitness data with circulating proteins in a sub-cohort at 10 years post-GDM. Specifically, we aimed to identify key circulating proteins associated with aerobic fitness and maximal muscle strength across five muscle groups, and to examine how these fitness-associated proteins relate to glycaemic status and cardiometabolic markers linked to T2D development in women after gestational diabetes. We further aimed to determine if these proteins could be altered by 6 weeks of training in an independent intervention study consisting of overweight/obese male participants. By focusing on circulating proteins, this study addresses the potential clinical utility of serum biomarkers, which are more accessible and scalable for cardiometabolic risk stratification, early detection, and monitoring of exercise intervention response.

## Methods

### Participants and recruitment

This study describes a sub-cohort of women (*n* = 38) from the PONCH (Pregnancy Obesity Nutrition Child Health) cohort [19] who, at 10 years post-GDM, underwent extensive physical tests [18], and provided serum samples for proteomic analyses [20]. Inclusion criteria for the PONCH 10-year visit were a pregnancy diagnosed with GDM (according to the 1999 WHO criteria [21]) between 2005 and 2009 in the Gothenburg area, and attendance of the 6-year study visit [19]. Pregnancy < 12 months prior to the 10-year visit and type 1-diabetes mellitus diagnosis were the exclusion criteria.

The clinical follow-up visits at 10 years included fasting venous blood sampling, oral glucose tolerance testing (OGTT), and body composition and anthropometric measures. All participants attending the 10-year clinical visit were invited to an additional visit for fitness testing; 45 participants agreed to and completed this fitness assessment, which was performed 48 ± 33 days after the 10-year clinical visit. Proteomic analysis was conducted on serum samples from 38 participants that met the criteria for validity, i.e. no haemolysis. The fitness variables consisted of measures of peak oxygen uptake (VO_2_peak), submaximal exercise fat oxidation, and isometric strength, as well as body composition measurements by dual-energy X-ray absorptiometry (DXA; GE Medical Systems, Madison, WI). Prescribed medication to the women who conducted the fitness testing included insulin (*n* = 1), metformin (*n* = 3), levothyroxine (*n* = 3), statin (*n* = 1), and blood pressure medication (*n* = 2). No instructions were given regarding medication intake on the day of the study.

### Study visits

The clinical visit and fitness assessment have previously been described [18]. In short, all visits were conducted in the morning following an overnight fast. The clinical visit at *Sahlgrenska University Hospital* included venous blood sampling, OGTT (a 2hour 75-g OGTT, with glucose and insulin measures every 30 minutes), and anthropometric measurements (height, weight, and hip and waist circumference). The fitness assessment at *Center for Health and Performance*, Gothenburg University included DXA body composition measurements (lean mass, fat mass, and android, gynoid and visceral fat mass) and aerobic fitness performance VO_2_peak, peak fat oxidation (PFO), fatmin (the intensity at which the lowest fat oxidation occur) and fatmax (the intensity at which PFO occurs) in the fasted state as previously published [18]. The aerobic fitness measurements were conducted using a graded exercise test to exhaustion on a cycle ergometer. Following a brief rest and a light breakfast, maximal isometric muscular strength of five muscle groups was assessed using dynamometers (David Health Solutions, Helsinki, Finland). The muscle groups tested were trunk flexion, knee flexion, knee extension, hip abduction, and hip adduction.

### Biochemical measurements

Blood samples were analysed for glucose, insulin, HbA1c, low-density lipoprotein (LDL), and high-density lipoprotein (HDL) at the certified Clinical Chemistry Laboratory at Sahlgrenska University Hospital (International Standard ISO 15189:2007), as previously described [18]. HOMA-IR was calculated as (fasting glucose × fasting insulin)/22.5, HOMA-B as (20 × fasting insulin)/(fasting glucose − 3.5), and Matsuda as 10 000/√(fasting insulin × fasting glucose) × (mean OGTT glucose × mean OGTT insulin). Participants were classified as having normal glycaemic tolerance (NGT), IGT, or T2D based on self-reported diabetes obtained by interview or a new diagnosis established at the study visit. Classification was based on fasting and 2-hour glucose levels after the OGTT, according to the 1999 WHO guidelines [21]. The IGT category included individuals with impaired fasting glucose (fasting glucose 6.1–6.9 mmol/L) and impaired glucose tolerance (2-hour glucose 7.8–11.0 mmol/L).

### Sample processing and LC-MS/MS analysis of PONCH study cohort

Proteomic analysis of the PONCH cohort was conducted as previously described [20] and performed in 2021. Briefly, 7 µl of serum per sample was used, with the 14 most abundant proteins depleted prior to processing following Easypep (Thermo Fisher) digestion protocol. The resulting digests were analysed by data-independent acquisition (DIA) on an Orbitrap Exploris 480 mass spectrometer coupled to an UltiMate 3000 RSLCnano System, using a 30-minute gradient. Library-free DIA data analysis was performed with Spectronaut (version 15) and further analysed with Perseus (version 1.6.15.0). Technical reproducibility and methodological robustness were confirmed by strong correlations between LC-MS/MS and enzyme-linked immunosorbent assay (ELISA) measurements of CRP, a known marker elevated in T2D [20, 22].

### Independent training intervention cohort

The methodology for the independent training intervention cohort has been described in depth elsewhere [9]. In brief, the study consisted of a 6-week training intervention, with a structed scheduled 1–4 days/week (average 3 days/week, attendance 99,8%) of supervised endurance exercise training, where exercise adaptations and health parameters were measured in untrained overweight or obese men without chronic disease (*n* = 28; age 28.4 ± 5.1y; BMI 30.4 ± 2.2 kg/m^2^). Participants underwent pre- and post-intervention assessments, including measurements of VO_2_peak and fasted venous blood samples. Body composition was evaluated using DXA. Body weight was monitored and energy intake adjusted throughout the study to ensure weight stability.

For the independent training cohort, the depletion serum samples were processed using a modified SP3-workflow, with proteomic analysis performed in 2025 and not published before. Peptide samples (500 µg) were loaded onto EvoTips. The LFQ four dimensional (4D) DIA analysis was conducted on a timsTOF HT mass spectrometer coupled to an Evosep One liquid chromatography system (Evosep) running the 30 samples per day (30SPD) method. The raw data was analysed using Spectronaut (Version 18.7).

### Statistical analysis

Exploratory proteomics analysis was performed in the retrospective cohort study. No methods were used to predetermine sample size. The analysts were not blinded to the clinical data. Downstream analyses were performed using R v4.5.1. Spearman correlation was used for correlation analyses between serum proteins and fitness variables, and serum proteins and clinical variables. VO_2_peak, PFO, and isometric strength variables were all adjusted for body weight before correlation analysis. Additionally, partial Spearman correlation analyses adjusting for age and medication use were performed to assess the robustness of the observed associations. Proteins with a Benjamini-Hochberg false discovery rate (FDR) < 0.1 were considered significant. The rationale for applying a relaxed FDR threshold was based on the use of unique participant material and the hypothesis-generating nature of the omics analysis. To further investigate these associations, participants were stratified into fitness-level groups based on aerobic capacity and muscle strength (as described below). Differences in protein levels across fitness-level groups were analysed using univariate linear regression, with fitness-level groups using aerobic capacity expressed to body weight and muscle strength expressed either as absolute strength or relative to lean mass. Comparisons across glycaemic status groups were analysed using multivariable linear regression models adjusted for age and medication use. In the independent training intervention cohort, pre- and post-measures were first analysed using non-parametric paired t-test (Wilcoxon signed-rank test). Linear mixed-effects model adjusted for age was subsequently applied to account for repeated measures and to confirm the robustness of the observed changes. For these analyses, *P* < 0.05 was considered significant.

The fitness-level groups were stratified as follows: The median of the sum of total muscle strength (all strength variables combined) was used to define the strength stratification, with values below the median classified as “low strength” and those above the median classified as “high strength”. Aerobic capacity was determined using normative values adjusted for age, based on Åstrand [23]. Participants with values below the Åstrand average were classified as having “low aerobic capacity,” while those at or above the average were classified as having “high aerobic capacity,” with the following thresholds: <40 years: average 34 ml/kg/min; ≥40 to < 50 years: average 32 ml/kg/min; ≥50 years: average 29 ml/kg/min. The participants were subsequently categorised into four groups: Group 1 (low aerobic capacity/low strength), Group 2 (low aerobic capacity/high strength), Group 3 (high aerobic capacity/low strength), and Group 4 (high aerobic capacity/high strength). One woman who lacked strength measures and was not categorised.

R v4.5.1 was used to create all figures except for the Venn-diagrams where Microsoft PowerPoint was used (v2411, Microsoft Corporation, Redmond, WA, USA).

## Results

### Participant characteristics

Table 1 describes the patient characteristics, including results from the fitness tests. No significant differences were observed in background characteristics such as parity or diabetic heredity between fitness-level groups, although ethnicity differed with more non-nordic women in the low fitness groups. Anthropometry and body composition differed between groups: BMI and fat percentage were highest in the low aerobic capacity groups (group 1 and group 2), whereas lean mass was the highest in the groups with high muscular strength (group 2 and group 4). Notably, all women with T2D were classified into group 1. HbA1c differed significantly across the fitness level groups, while other glycaemic measures showed no significant between-group differences. As per design, all fitness variables differed significantly between groups, except for fatmax, for which a non-significant trend was observed.

**Table 1:** Participant characteristics of the women with a history of GDM, mean (± SD)

		Fitness-level groups				
	Overall	Group 1 Low aerobic capacity, low strength	Group 2 Low aerobic capacity, high strength	Group 3 High aerobic capacity, low strength	Group 4 High aerobic capacity, high strength	*p*
*n*	38	14	10	4	9	
Ethnicity % (Swedish/Non-Nordic)	65/35	**25/67**	**25/33**	**17/0**	**33/0** ^**#**^	**0.017**
Parity % (0/1/2+)	42/40/18	25/33/83	43/20/0	13/13/0	19/33/17	0.230
Diabetes heredity % (No/Yes)	49/51	33/44	39/17	11/11	17/28	0.480
Glycaemic Tolerance % (NGT/IGT/T2D)	61/26/13	32/20/100	32/30/0	4/30/0	32/20/0	0.052
Age (years)	43.7 (4.6)	**45.7 (4.7)**	42.4 (3.9)	44.6 (6.6)	**41.8 (3.7)** ^**#**^	0.103
BMI (kg/m^2^)	26.7 (4.7)	**27.2 (4.5)**	**30.3 (3.8)**	**20.0 (2.6)**	**25.0 (2.4)**	**0.002**
Lean mass (kg)	41.6 (5.2)	**38.3 (4.3)**	**45.0 (2.9)**	**36.0 (1.4)**	**44.5 (4.1)** ^**#**^	**< 0.001**
Fat percentage (%)	37.0 (6.7)	**39.7 (5.4)**	**41.6 (3.8)**	**28.8 (5.3)**	**32.5 (5.4)** ^**#**^	**< 0.001**
HbA1c (mmol/mol)	35.9 (8.9)	**40.9 (13.0)**	**32.8 (3.0)**	**31.8 (3.0)**	**33.2 (1.8)** ^**#**^	**0.019**
Fasting glucose (mmol/L)	5.9 (1.3)	6.4 (1.9)	5.5 (0.6)	6.0 (0.5)	5.4 (0.5)	0.312
HOMA-IR	2.9 (2.4)	3.4 (2.9)	3.6 (2.6)	1.8 (1.6)	1.6 (0.5)	0.175
Glucose AUC (mmol*h/L)	15.5 (3.9)	17.6 (4.3)	14.9 (3.8)	15.6 (3.4)	13.4 (3.2)	0.161
Leg extension (Nm)	223 (73)	**157 (53)**	**260 (30)**	**219 (50)**	**288 (56)** ^**#**^	**< 0.001**
Leg flexion (Nm)	181 (53)	**137 (42)**	**190 (32)**	**169 (26)**	**244 (22)** ^**#**^	**< 0.001**
Leg abduction (Nm)	191 (45)	**156 (29)**	**215 (25)**	**159 (30)**	**231 (35)** ^**#**^	**< 0.001**
Leg adduction (Nm)	210 (51)	**167 (42)**	**238 (26)**	**180 (28)**	**261 (21)** ^**#**^	**< 0.001**
Abdominal flexion (Nm)	89 (26)	**70 (19)**	**99 (26)**	**73 (9)**	**115 (12)** ^**#**^	**< 0.001**
VO_2_peak (ml/kg/min)	30.2 (6.7)	**25.2 (4.3)**	**28.5 (2.7)**	**39.8 (2.9)**	**36.3 (4.9)** ^**#**^	**< 0.001**
Peak fat oxidation (g/min)	0.2 (0.1)	**0.2 (0.1)**	**0.3 (0.1)**	**0.2 (0.1)**	**0.3 (0.1)** ^**#**^	**< 0.001**
Fatmax (%)	46.5 (8.0)	44.2 (7.9)	46.5 (9.0)	51.5 (4.0)	50.8 (7.7)	0.201
Fatmin (%)	84.2 (9.9)	**78.8 (9.9)**	**84.7 (10.2)**	**91.4 (5.2)**	**90.9 (6.0)** ^**#**^	**0.009**

*P* value for Kruskal-Wallis ranked sum test between groups (*P*< 0.05 in bold). ^#^*P* < 0.05 for Mann-Whitney U test for continuous and Fisher’s extract test for categorical variables between group 1 and group 4. Bold indicates statistical significance. One participant lacked strength measurements and was not categorised

AUC, area under the curve; BMI, body mass index; Fatmax, maximal fat oxidation intensity; Fatmin, minimal fat oxidation intensity; HbA1c, glycated haemoglobin; HOMA-IR, homeostatic model assessment of insulin resistance; IGT, impaired glucose metabolism; Nm, Newton meters; NGT, normal glucose tolerance; VO_2_peak, peak oxygen uptake; T2D, type 2-diabetes

### Identification of fitness related proteins

**Fig. 1:**
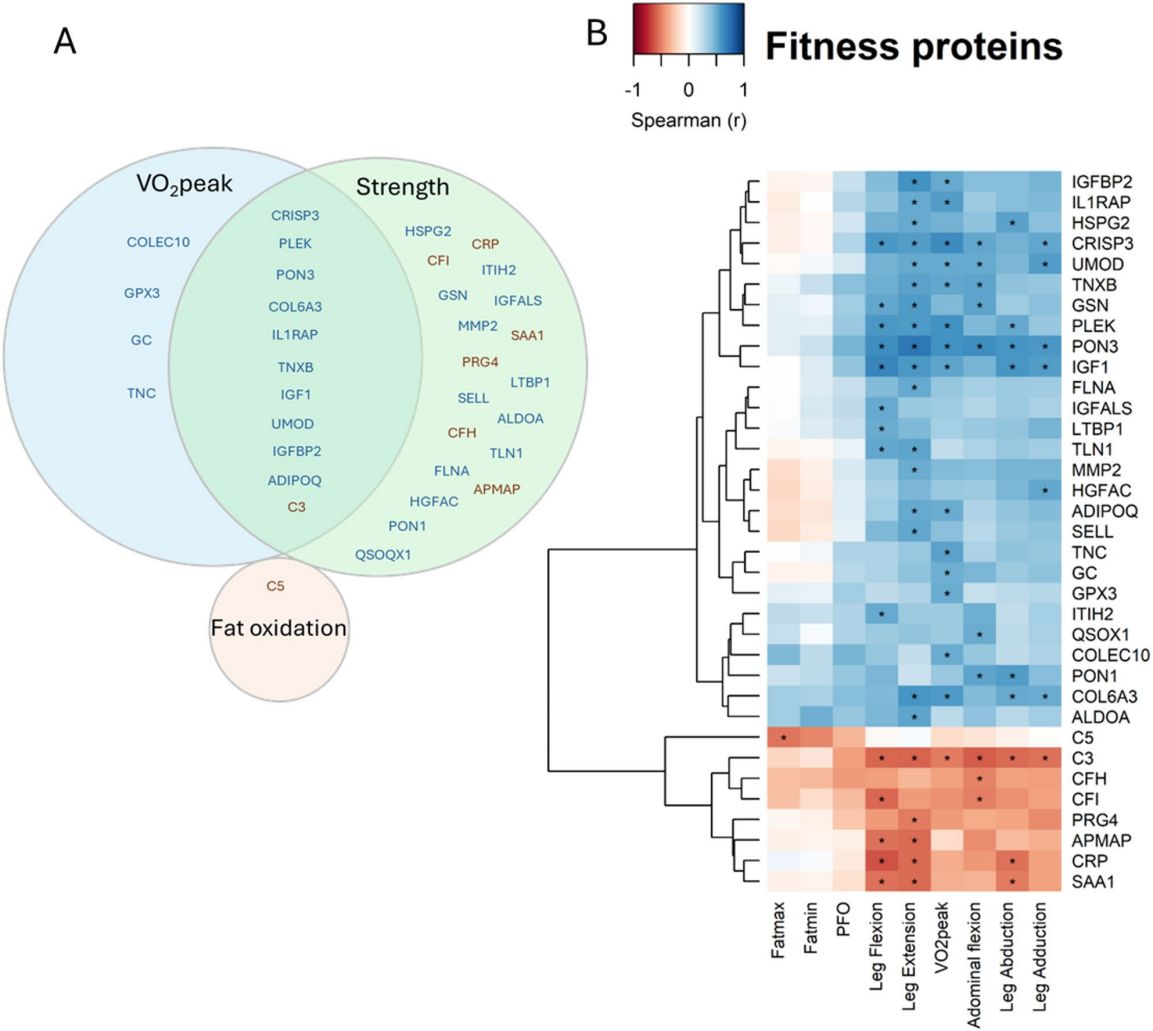
Fitness proteins. **A** Venn-diagram illustrating the type of fitness measure the identified fitness proteins associated with (blue text indicates a positive association and brown negative), **B** heatmap illustrating the individual correlations between proteins and specific fitness measures. Stars indicate significant relationships (FDR < 0.1)

Thirty-five proteins correlated with at least one fitness measurement (Fig. 1) and are hereafter referred to as fitness proteins. Relative VO_2_peak (ml/kg/min) was significantly associated with higher circulating levels of 14 proteins (CRISP3, COL6A3, PLEK, PON3, IL1RAP, TNC, IGF1, TNXB, UMOD, IGFBP2, ADIPOQ, COLEC10, GPX3, GC) and lower circulating levels of one protein (C3). Of these 15 proteins, 11 also correlated with at least one strength variable. For fat oxidation, only C5 was significantly associated, showing a negative correlation with fatmax. Thirty proteins associated with strength measurements, of which seven showed positive associations (PON3, GSN, IGF1, COL6A3, PLEK, CRISP3, UMOD) and three negative associations (C3, CRP, SAA1) with at least three strength variables. Notably, PON3, IGF1, CRISP3, COL6A3, and C3 emerged as proteins of particular interest, showing the largest and most consistent effect sizes across fitness outcomes. After adjustment for age and medication, 21 of the identified 35 fitness proteins remained significant, including all five of the proteins of interest (Fig. S1).

**Fig. 2:**
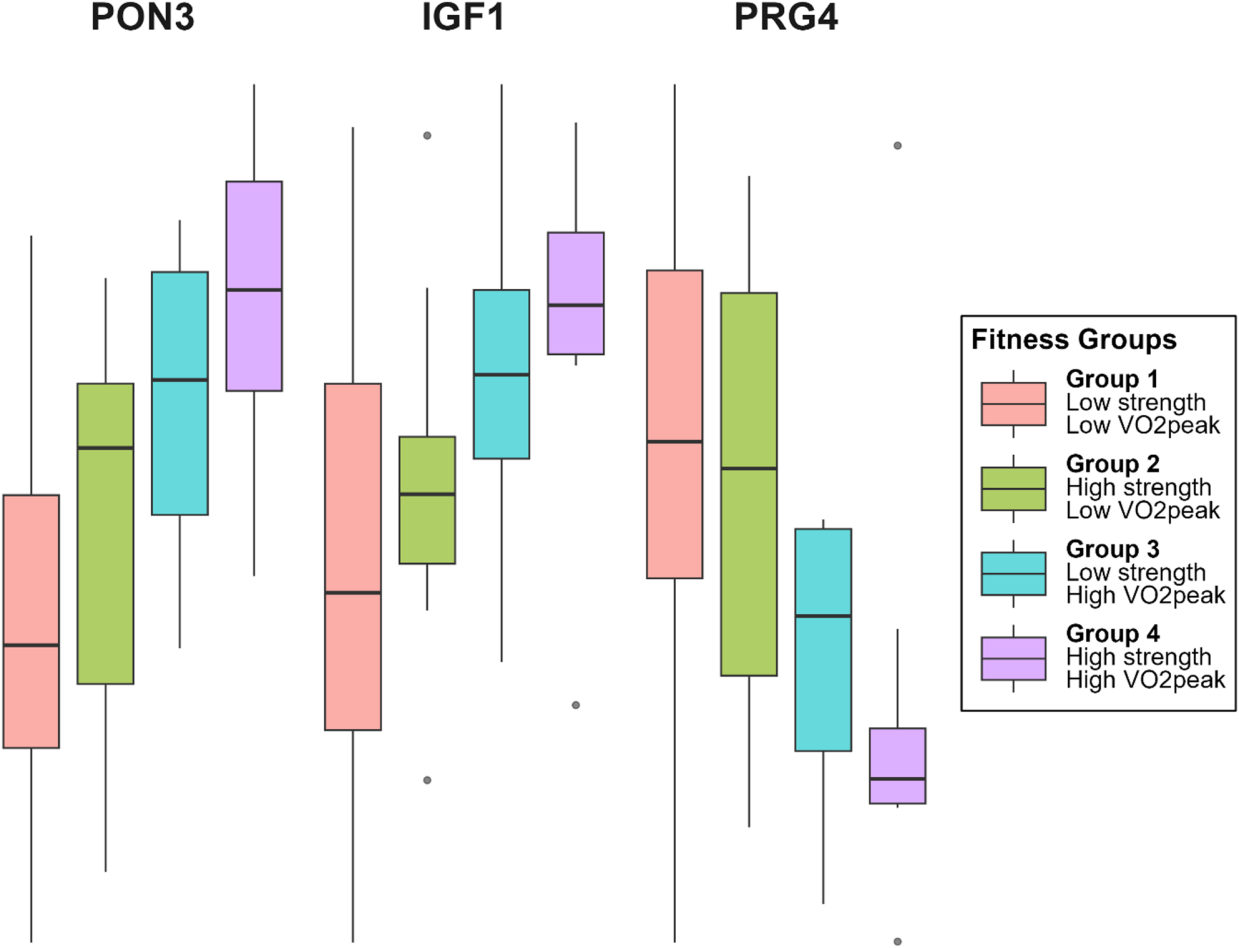
Fitness-associated proteins across four fitness-level groups. Shown are proteins that differed between the lowest and highest fitness group (Group 4 vs. Group 1; univariate linear regression *p* < 0.05) and remained significant after Benjamini-Hochberg correlation (FDR < 0.1), with a consistent monotonic change across all groups. Boxplot show median (central line), interquartile range (Q1-Q3), whiskers (1.5 x IQR) and outliers (points).

A univariate linear regression analysis, adjusted for body weight, identified significant differences in IGF1 (FDR 0.072, *p* = 0.007), PON3 (FDR 0.033, *p* < 0.001), and PRG4 (FDR 0.072, *p* = 0.014) between group 1 (least fit) and 4 (fittest) (Fig. 2). Fitness measures are commonly normalised to body weight; however, normalisation to lean mass may provide additional insight into performance relative to muscle mass. After further adjustments for lean mass, only PON3 remained significantly different between these groups (FDR 0.051, *p* < 0.001).

### Fitness proteins versus clinical variables

**Fig. 3:**
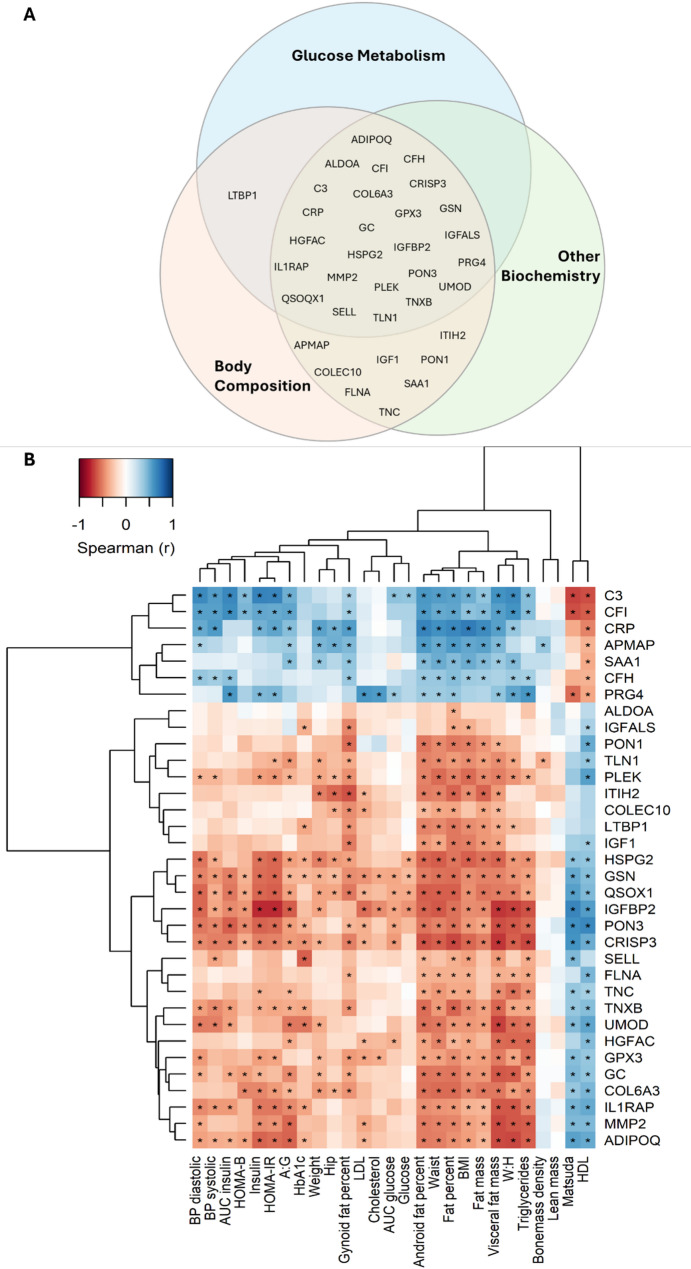
Fitness proteins vs. clinical variables. **A** clinical variables grouped in Venn-diagram to illustrate correlation with fitness proteins, **B** heatmap depicts the correlation between specific fitness proteins and specific clinical variables. Stars indicate significant relationship (FDR < 0.1)

Figure 3 illustrates the correlation between the fitness proteins and clinical variables. All fitness proteins, except C5, correlated with at least one clinical variable. Among the 34 fitness proteins, all were associated with body composition and/or anthropometric measures. Fat percentage demonstrated the strongest correlation, being significantly linked to all 34 proteins (7 positively and 27 negatively associated). No significant correlations were found between fitness proteins and absolute lean mass. Most fitness proteins were also correlated with glucose metabolism variables and cardiometabolic markers. Many of these associations persisted after adjustment for age and medication use (Fig. S2).

Among the five proteins identified of particular interest, PON3, CRISP3, and C3 were consistently associated with blood lipids and blood pressure; PON3, CRISP3, C3, and COL6A3 with insulin resistance; and all five proteins (PON3, CRISP3, C3, COL6A3, IGF1) with central adiposity. These associations remained significant after adjustment for age and medication use.

### Fitness proteins in glycaemic groups

To further investigate the relationship between fitness proteins and glucose metabolism, a univariate linear regression analysis was performed across the glucose tolerance groups. Among the 35 fitness proteins, only five differed significantly between the T2D and NGT groups. Four proteins were present at lower levels, while one protein was present at higher levels in the T2D group compared with the NGT group (Table 2). These five proteins remained significantly associated after adjustment for age and medication (Table S1).

**Table 2:** Linear regression comparing glycaemic groups versus fitness-associated proteins

Protein	NGT	IGT	T2D	FDR
*n*	23	10	5	
C3		0.01	0.27**	0.070
CRISP3		−0.20	−0.82*	0.070
GPX3		−0.01	−0.49*	0.070
GSN		−0.08	−0.36**	0.046
MMP2		0.01	−0.42*	0.070
QSOX1		−0.17	−0.36*	0.074

Shown are regression values for significant proteins, expressed as coefficient estimates, that differed significantly between NGT and T2D groups and remained significant after Benjamini-Hochberg (FDR < 0.1), with a consistent monotonic change across all groups. *p < 0.05 **p < 0.01 compared to NGT group

FDR, false discovery rate; IGT, impaired glucose tolerance; NGT, normal glucose tolerance; T2D type-2 diabetes

### Exercise responsiveness in an independent training intervention cohort

**Fig. 4:**
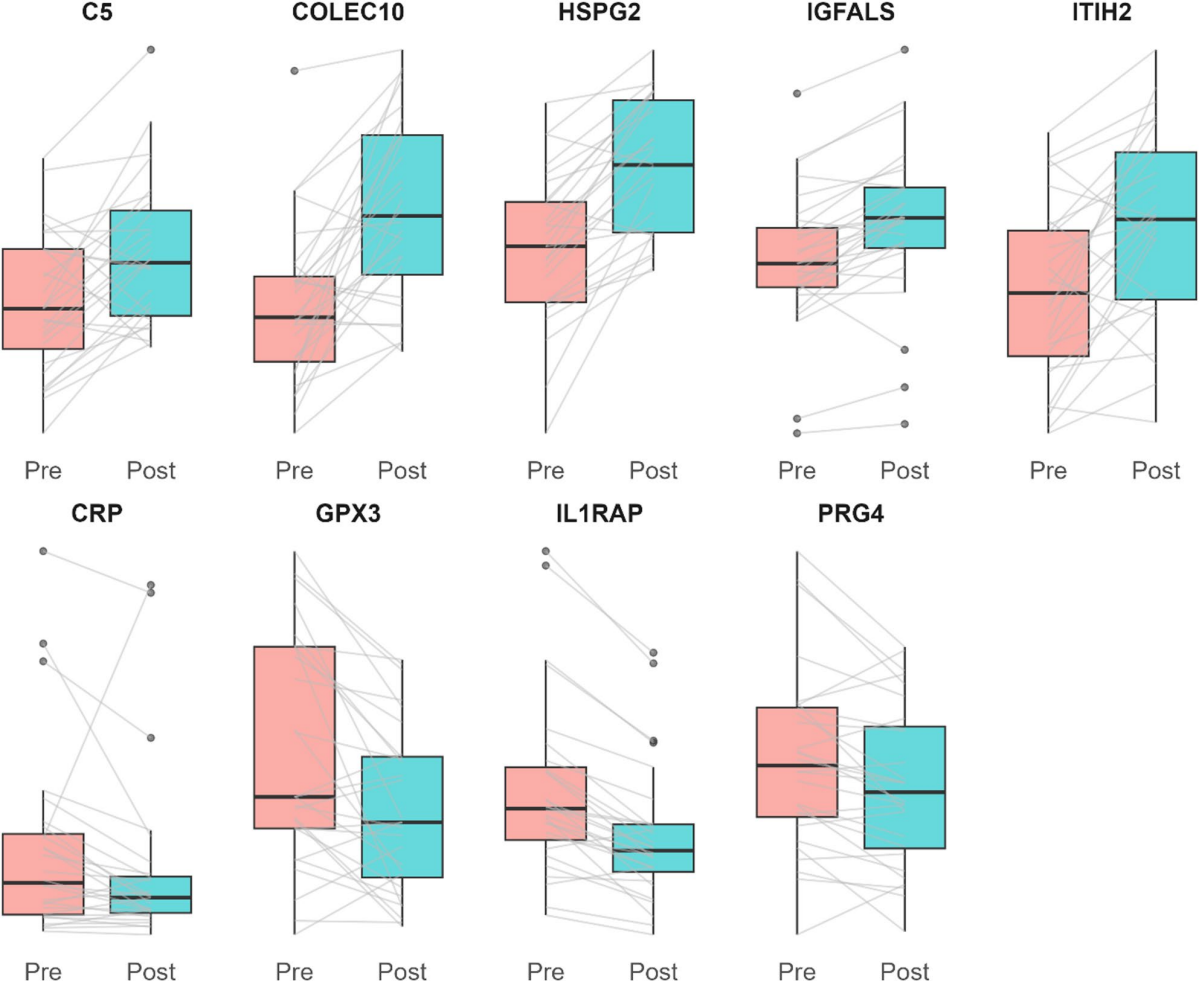
Fitness-associated proteins in the independent training cohort, pre- and post-intervention. Shown are proteins that changed pre- and post-intervention following a structured and supervised 6-week aerobic exercise intervention in an independent cohort of men (Wilcoxon signed-rank test; Benjamini-Hochberg FDR-adjusted *p* < 0.05). Boxplots show median (central line), interquartile range (Q1-Q3), whiskers (1.5 x IQR) and outliers (points)

To investigate whether the identified fitness proteins change with exercise training, we sampled fasting serum from an independent cohort of untrained overweight or obese male participants who had performed a rigorous 6-week aerobic exercise intervention. The participants increased their VO_2_peak by 7% during the intervention (Table 3). As instructed in the study, the participants did not change their weight but showed a small shift in body composition towards lower body fat percentage. The participants were metabolically healthy at the start of the intervention and no change was seen in blood glucose, HOMA-IR, or blood lipids. Proteomics analysis revealed that nine of the 35 fitness proteins changed significantly following the training intervention (Fig. 4). Five proteins showed increased circulating levels (C5, COLEC10, HSPG2, IGFALS, ITIH2) and four showed decreased levels (CRP, GPX3, IL1RAP, PRG4). Of these nine fitness-associated proteins, six (COLEC10, HSPG2, IGFALS, ITIH2, CRP, PRG4) changed in the same direction as observed in the associations of the post-GDM PONCH cohort. Following adjustment for age, all differences remained significant, except for CRP (Table S2).

**Table 3:** Pre- and post-characteristics of an independent male cohort performing a 6-weeks aerobic exercise intervention

	Pre intervention	Post intervention	Difference	*p*
VO_2_peak (ml/kg/min)	38.8	41.7	2.9	**< 0.001**
BMI (kg/m^2^)	30.7	30.5	−0.1	0.17
Fat (%)	**32.8**	**31.9**	**−0.9**	**< 0.001**
Lean mass (kg)	**64.1**	**64.8**	**0.7**	**0.002**
Fat mass (kg)	**33.2**	**32.3**	**−0.9**	**0.01**
Glucose (mmol/L)	5.5	5.5	0.0	0.77
Insulin (mU/L)	11.2	9.7	−1.5	0.16
HOMA-IR	2.7	2.4	−0.4	0.23
Triglycerides (mmol/L)	1.3	1.4	0.2	0.24
Cholesterol (mmol/L)	5.2	5.0	−0.2	0.07
HDL (mmol/L)	1.3	1.3	0.0	0.66
LDL (mmol/L)	3.6	3.4	−0.2	0.13

Wilcoxon signed-rank test (*P* < 0.05 in bold)

BMI, Body mass index; HDL, High-density lipoprotein; HOMA-IR, Homeostatic model assessment of insulin resistance; LDL, Low-density lipoprotein; VO_2_peak, Peak oxygen uptake

## Discussion

This study identified serum proteins associated with physical fitness, measured as aerobic capacity and maximal muscle strength, in women with a history of GDM, a population at elevated risk for cardiometabolic disease, including T2D and cardiovascular disease. To our knowledge, this is the first study to assess the serum proteome in relation to both aerobic and muscular fitness in a high-risk cohort. We identified 35 proteins that correlated with at least one fitness variable, with most proteins also showing associations with clinical measures of metabolic health. These findings suggest that specific circulating proteins may serve as biomarkers reflecting fitness status in this population. While mechanistic conclusions cannot be drawn from this cross-sectional analysis, the biological functions of the identified proteins hint at potential molecular pathways linking fitness to key cardiometabolic health, including roles in metabolic regulation, immune function, complement activation, oxidative stress defence, and extracellular matrix remodelling.

We identified 35 proteins associated with fitness variables 10 years after pregnancy. Eleven proteins correlated with both VO_2_peak and muscle strength, 19 with muscle strength only, four with VO_2_peak only, and one with fat oxidation. Most proteins were positively associated with higher fitness levels, while eight showed inverse relationships. Candidate proteins showing the strongest and most consistent associations included PON3, IGF1, CRISP3, COL6A3, and C3 which were linked to both aerobic fitness and multiple measures of maximal muscle strength. Six of the 35 fitness-associated proteins were responsive to exercise and changed in the same direction as observed in the associations of the post-GDM PONCH cohort, following an aerobic training intervention in an independent cohort of untrained men, supporting their sensitivity to exercise.

Previous research in this post-GDM cohort demonstrated that both aerobic fitness and muscle strength were independently associated with glucose tolerance and insulin sensitivity [18]. In the current analysis, where we stratified participants into subgroups based on levels of aerobic fitness and muscle strength, we found that all women with T2D were classified into the group with both the lowest aerobic fitness and the lowest muscle strength. This finding highlights the potential clinical relevance of maintaining both aspects of fitness in relation to cardiometabolic risk, including the prevention or progression of T2D. Importantly, the current proteomic data provide new insights into the molecular pathways that may underlie the links between physical fitness, metabolic regulation, and broader cardiometabolic health.

The fitness-associated proteomic profile mapped to several key biological functions, each potentially linked to various aspects of physical performance and metabolic regulation. Proteins involved in metabolic regulation – including IGF1, IGFBP2, ADIPOQ, ALDOA, APMAP, and IGFALS – were positively associated with fitness, reflecting their established roles in promoting glucose uptake, insulin sensitivity, and energy metabolism [24–27]. Interestingly, some metabolic proteins, like IGF1 and IGFALS, showed strong links to both aerobic capacity and muscle strength, suggesting that systemic metabolic control may underpin both cardiovascular and muscular fitness and thereby influence cardiometabolic risk profiles.

Proteins related to immune and inflammatory pathways, including CRP, IL1RAP, PLEK, CRISP3, SAA1, SELL, and complement components (C3, CFI, CFH, COLEC10), showed consistent associations with fitness. Higher aerobic capacity and greater muscle strength were associated with lower levels of pro-inflammatory and complement proteins, supporting the role of physical fitness in dampening systemic inflammation and innate immune activation [28–30]. Notably, C3 levels inversely correlated with fitness status, consistent with prior findings that reduced C3 is a marker of higher aerobic fitness and lower metabolic disease risk [31, 32]. Complement activation may therefore represent a mechanistic link between low fitness and elevated cardiometabolic disease risk.

Proteins associated with oxidative stress regulation, such as PON3, PON1, QSOX1, and GPX3, were also positively linked to fitness. Regular exercise is known to enhance antioxidant defences, and higher levels of these proteins could reflect a better capacity to neutralise reactive oxygen species generated during muscle work and mitochondrial metabolism [33–35], processes implicated in insulin resistance and vascular dysfunction [36]. These findings are consistent with previous work showing that regular exercise enhances endogenous antioxidant defence systems, including enzymatic activity of glutathione peroxidases and paraoxonases [37]. Interestingly, PON3 was strongly associated with both aerobic and muscular fitness, suggesting it may be an important biomarker of systemic resilience against oxidative stress across multiple domains of physical function.

We also identified several proteins involved in cell structure and extracellular matrix (ECM) remodelling, such as COL6A3, TNXB, HSPG2, MMP2, TLN1, FLNA, LTBP1, ITIH2, TNC, and GSN, which were associated with muscle strength. This observation is biologically plausible, given that strength training and muscle loading induce mechanical strain, ECM remodelling, and adaptation of connective tissues [38–40]. COL6A3, TNXB, and TNC have been implicated in tissue elasticity, fibrosis prevention, and vascular remodelling, processes essential for maintaining muscle integrity and function.

Among the proteins most strongly linked to fitness, PON3, IGF1, CRISP3, COL6A3, and C3 stood out. IGF1 is well-known for its dual role in supporting muscle hypertrophy and metabolic regulation [25, 41, 42]. CRISP3 and COL6A3, while less studied in exercise contexts, may represent novel biomarkers capturing inflammation-related and structural adaptations, respectively. Together, these proteins may provide insight into metabolic, inflammatory, and structural processes linked to cardiometabolic risk.

Furthermore, when stratifying participants based on combined aerobic and muscle strength fitness levels, only IGF1, PON3, and PRG4 remained consistently associated, suggesting that these proteins may reflect a more global, integrative index of fitness status.

In addition to fitness measures, nearly all fitness-associated proteins also correlated with body composition – particularly fat mass and visceral adiposity – while few were linked to lean mass. Fat percentage was significantly associated with 34 of the 35 proteins, highlighting adiposity as a major modifier of circulating protein levels. Several proteins, including ADIPOQ, APMAP, and CRP, are known to reflect adipose tissue metabolism and inflammation, and have been previously linked to insulin resistance and metabolic syndrome [43–45]. These findings suggest that physical fitness and adiposity influence overlapping molecular pathways and may jointly contribute to metabolic risk in women after GDM. The strong correlations with glucose homeostasis and insulin sensitivity further underscore their potential as biomarkers of cardiometabolic health.

In our independent training intervention cohort, composed of overweight or obese but otherwise metabolically healthy men, six (COLEC10, HSPG2, IGFALS, ITIH2, CRP, PRG4) of the 35 fitness-associated proteins changed in the expected direction after only six weeks of aerobic training, despite no significant weight loss. This finding emphasises that many of these circulating proteins respond primarily to changes in fitness and cardiorespiratory capacity, independent of weight loss.

### Strengths and limitations

Strengths of this study include the use of a well-characterised post-GDM cohort with extensive clinical phenotyping, including fasting and OGTT-derived glucose control measures across all glucose tolerance categories. Unlike many diabetes studies, the cohort was relatively young and free from major comorbidities [20], reducing confounding factors. Proteomics analysis was conducted using robust LC-MS/MS workflows with high technical reproducibility, validated against ELISA-based inflammatory markers. The comprehensive fitness assessment – including objective strength measurements from multiple muscle groups rather than just grip strength as seen in most other studies – provides a broader picture of musculoskeletal function.

Exercise responsiveness of fitness-associated proteins was further assessed in an independent training cohort. Notably, these men were untrained and had a pre-intervention VO_2_peak (38.8 ml/kg/min) comparable to the high-aerobic-capacity groups in our post-GDM cohort (Groups 3–4: 36.9–39.8 ml/kg/min), increasing to 41.7 ml/kg/min post-intervention. Several proteins (C5, COLEC10, HSPG2, IGFALS, ITIH2, CRP, GPX3, IL1RAP, PRG4) were confirmed to be important signatures of exercise capacity across the cohorts. However, the training cohort differed by sex, age, and metabolic status, in addition to the intervention being relatively short, which may limit generalisability. The intervention’s focus on aerobic exercise leaves the effects of resistance training on these proteins to be explored in future studies.

Limitations include challenges with VO_2_peak testing, as maximal tests can be difficult for untrained individuals. Submaximal protocols, like the Ekblom-Bak test, may enhance feasibility in future cohorts. Additionally, the number of women with complete fitness and proteomics data, particularly those with T2D, was limited, reducing statistical power for some subgroup analyses. To address these limitations and to validate the fitness-associated proteins found in this study, work is ongoing in a larger, independent post-GDM cohort with longitudinal fitness and proteomics measurements [46].

### Conclusion

This study identified a panel of circulating proteins linking aerobic fitness and muscle strength to key cardiometabolic pathways, including metabolic regulation, inflammation, complement activation, oxidative stress, and structural remodelling in women at risk for T2D after GDM. By focusing on serum rather than muscle tissue – an approach more commonly used in previous proteomic studies of fitness – our findings increase translational relevance, as circulating biomarkers are more accessible and scalable for risk stratification, early detection, and monitoring of exercise responses in at-risk populations. Together, these findings offer new insights into the molecular basis of physical fitness and may guide the development of novel biomarkers for identification of metabolic dysfunction and targets for exercise interventions. Further studies, particularly intervention studies incorporating resistance training, are warranted to confirm and expand on these findings.

## Supplementary Information

Below is the link to the electronic supplementary material.

Supplementary Material 1

## Data Availability

MS proteomics data are available at the MassIVE repository, a ProteomeXchange Consortium partner, with the dataset identifier MSV000092252. The clinical and fitness data used during the current study are available from the corresponding author on reasonable request.

## Abbreviations

ADIPOQ: Adiponectin
ALDOA: Aldolase A
APMAP: Adipocyte plasma membrane associated protein
AUC: Area under the curve
BMI: Body mass index
C3: Complement component 3
C5: Complement component 5
CFH: Complement factor H
CFI: Complement factor I
COL6A3: Collagen type VI alpha 3 chain
COLEC10: Collectin sub-family member 10
CRISP3: Cysteine-rich secretory protein 3
CRP: C-reactive protein
DIA: Data-independent acquisition
DXA: Dual-energy X-ray absorptiometry
FDR: False discovery rate
GC: Vitamin D-binding protein (group-specific component)
GDM: Gestational diabetes mellitus
GPX3: Glutathione peroxidase 3
GSN: Gelsolin
HbA1c: Glycated haemoglobin
HDL: High-density lipoprotein
HOMA-B: Homeostatic model assessment of β-cell function
HOMA-IR: Homeostatic model assessment of insulin resistance
HSPG2: Heparan sulfate proteoglycan 2 (Perlecan)
IGF1: Insulin-like growth factor 1
IGFALS: Insulin-like growth factor-binding protein acid labile subunit
IGFBP2: Insulin-like growth factor binding protein 2
IGT: Impaired glucose tolerance
IL1RAP: Interleukin-1 receptor accessory protein
ITIH2: Inter-alpha-trypsin inhibitor heavy chain H2
LC-MS/MS: Liquid chromatography tandem mass spectrometry
LDL: Low-density lipoprotein
LTBP1: Latent transforming growth factor beta-binding protein 1
MMP2: Matrix metallopeptidase 2
MS: Mass spectrometry
NGT: Normal glucose tolerance
Nm: Newton meter
OGTT: Oral glucose tolerance test
PFO: Peak fat oxidation
PLEK: Pleckstrin
PON1: Paraoxonase 1
PON3: Paraoxonase 3
PRG4: Proteoglycan 4 (lubricin)
QSOX1: Quiescin sulfhydryl oxidase 1
SAA1: Serum amyloid A1
SELL: L-selectin
T2D: Type 2-diabetes
TNC: Tenascin C
TNXB: Tenascin-X
TLN1: Talin 1
UMOD: Uromodulin
VO2peak: Peak oxygen uptake
