# Supplementary material for: Circulating protein biomarkers of physical fitness associated with cardiometabolic risk in women after gestational diabetes: a PONCH study
Source: Cardiovasc Diabetol. 2026 Feb 27;25:114. doi: 10.1186/s12933-026-03120-4 (PMC13049936; doi:10.1186/s12933-026-03120-4)
Supplement: Supplementary file 1 — Supplementary Material 1 [file 12933_2026_3120_MOESM1_ESM.docx]

**Supplemental Material 1**

**Table of content:**

- **Supplementary Tables**
  - Table S1. Linear regression results for the fitness-associated proteins showing differences across glycaemic status groups, adjusted for age and medication use.
  - Table S2. Exercise responsiveness of fitness-associated proteins showing significant changes after aerobic training, identified using age-adjusted linear mixed-effect model in an independent training intervention cohort.
- **Supplementary Figures**
  - Figure S1. Heatmap illustrating age and medication adjusted partial Spearman correlation between proteins and fitness variables.
  - Figure S2. Heatmap illustrating age and medication adjusted partial Spearman correlation between fitness-associated proteins and clinical variables.

**Table S1**. Linear regression results for fitness-associated proteins showing differences across glycaemic groups, adjusted for age and medication use.

| **Protein** | **NGT** | **IGT** | **T2D** | **FDR** |
| --- | --- | --- | --- | --- |
| *n* | *23* | *10* | *5* |  |
| **C3** |  | -0,02 | 0,26* | 0.070 |
| **CRISP3** |  | -0,10 | -0,81* | 0.070 |
| **GPX3** |  | 0,04 | -0,47* | 0.070 |
| **GSN** |  | -0,07 | -0,35** | 0.046 |
| **MMP2** |  | 0,04 | -0,41* | 0.070 |
| **QSOX1** |  | -0,15 | -0,32* | 0.074 |

Shown are regression values for significant proteins, expressed as coefficient estimates, that differed significantly between NGT and T2D groups and remained significant after Benjamin-Hochberg (FDR <0.1), with a consistent monotonic change across all groups. Models were adjusted for age and medication use. *p < 0.05, **p < 0.01 compared with the NGT group. FDR indicates Benjamin-Hochberg false discovery rate. IGT, impaired glucose tolerance; NGT, normal glucose tolerance; T2D type-2 diabetes.

**Table S2**. Exercise responsiveness of fitness-associated proteins showing significant changes after aerobic training, identified using age-adjusted linear mixed-effect model in an independent training intervention cohort.

| **Protein** | **Estimate (post-pre)** | **P** | **FDR** |
| --- | --- | --- | --- |
| **C5** | 2885 | 0.0012 | 0.006 |
| **COLEC10** | 179 | <0.001 | <0.001 |
| **HSPG2** | 91 | <0.001 | <0.001 |
| **IGFALS** | 1407 | <0.001 | <0.001 |
| **ITIH2** | 4207 | 0.0002 | 0.001 |
| **GPX3** | -1187 | 0.0011 | 0.006 |
| **IL1RAP** | -313 | <0.001 | <0.001 |
| **PRG4** | -407 | 0.0031 | 0.014 |

Exercise responsiveness was assessed as pre- and post-training change using linear mixed-effect model with participant as a random effect and adjustment for age. The estimate represents the fixed-effect coefficient for time (post vs pre), indicating the direction and magnitude of change following training. Only proteins remaining significant after Benjamin-Hochberg (FDR < 0.05) are shown. P-values represent fixed effects for time (post vs pre).


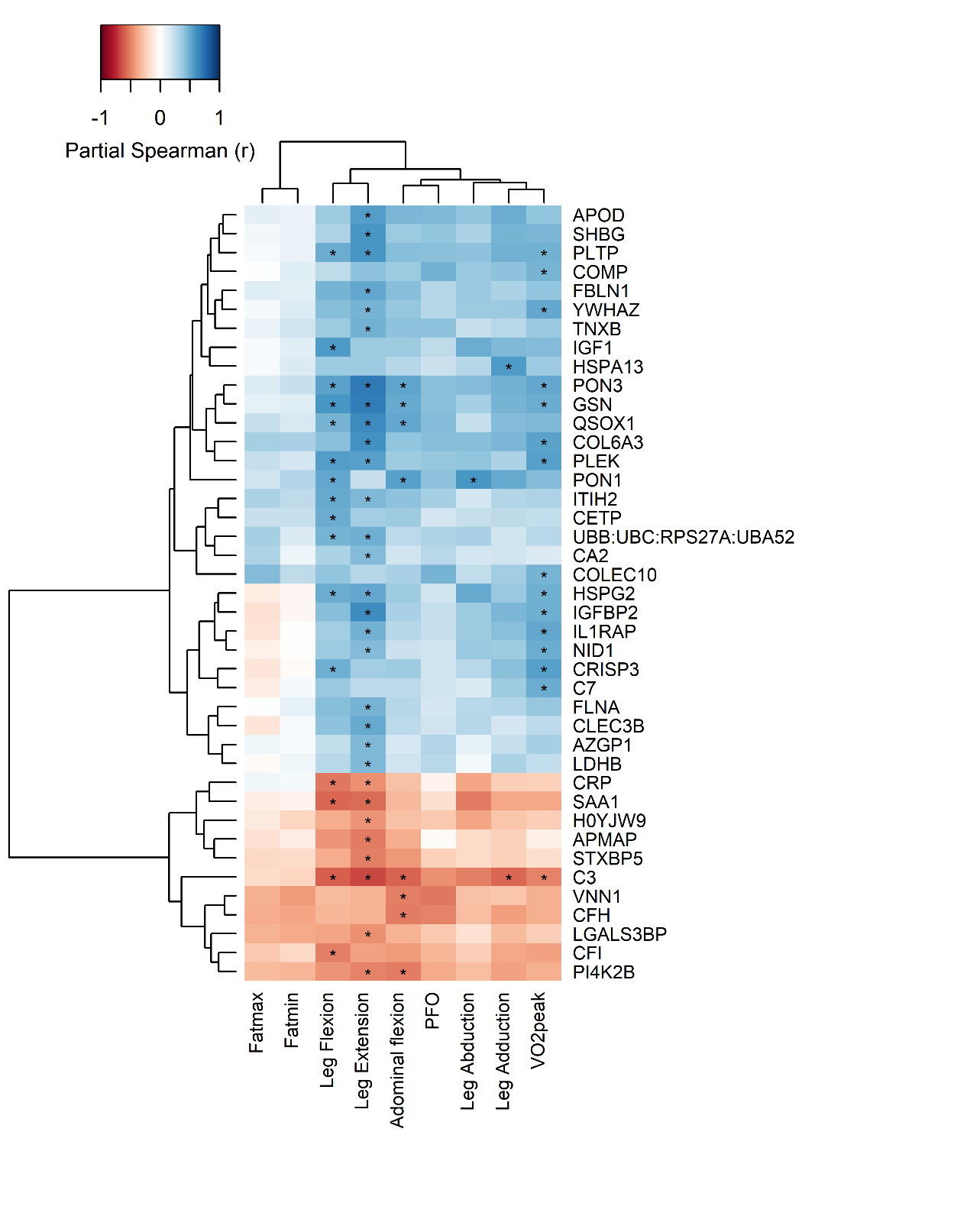


**Figure S1**. Heatmap illustrating partial Spearman correlation between circulating proteins and fitness variables. Correlations were adjusted for age and medication use. Asterisks denote statistically significant association after Benjamin-Hochberg correction (FDR < 0.1).

**
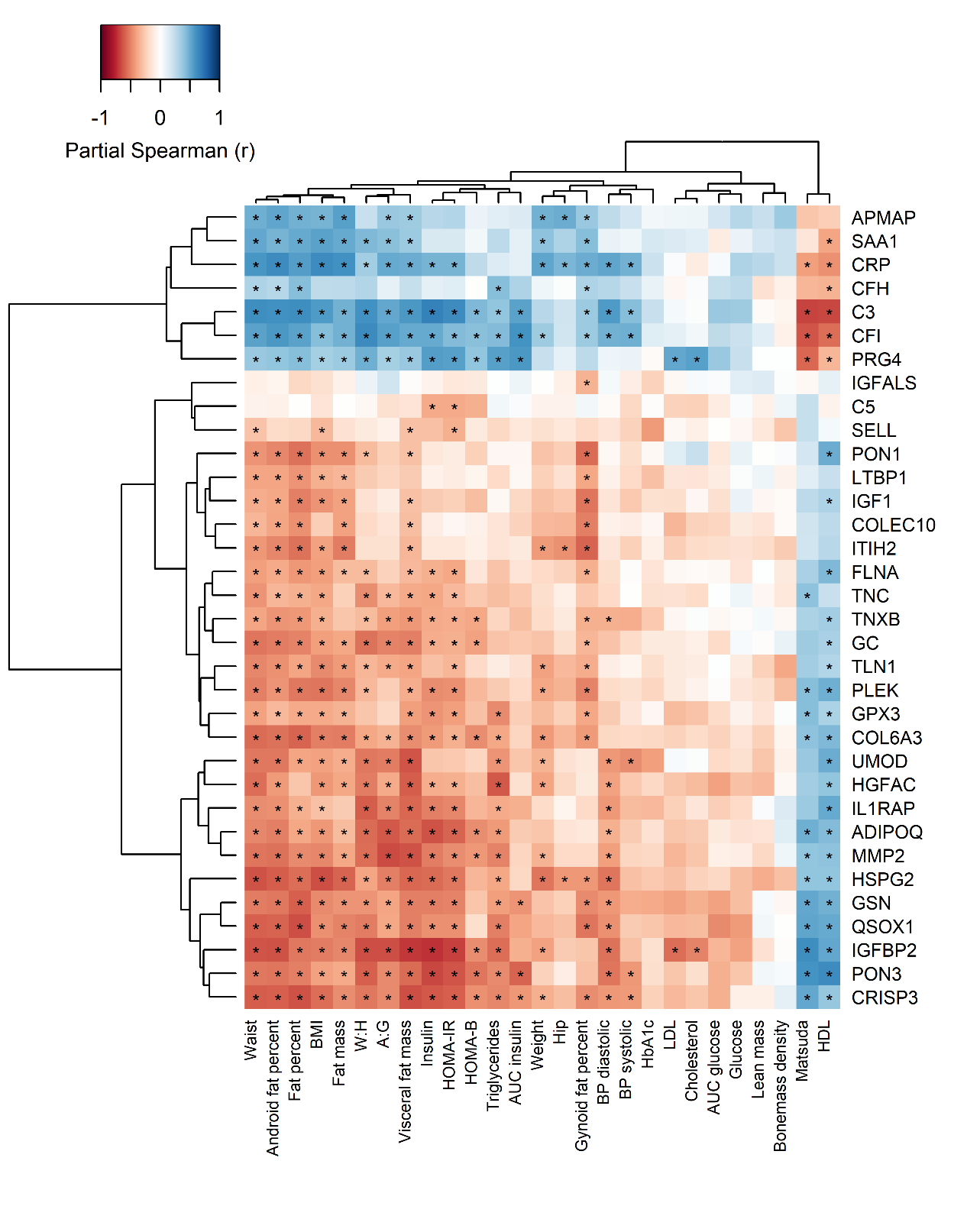
**

**Figure S2**. Heatmap illustrating partial Spearman correlation between circulating fitness-associated proteins and clinical variables. Correlations were adjusted for age and medication use. Asterisks denote statistically significant association after Benjamin-Hochberg correction (FDR < 0.1).
